# Syndrome of Inappropriate Anti-diuretic Hormone (SIADH) in Non-small Cell Lung Cancer (NSCLC) Following Agent Orange Exposure: A Case Report

**DOI:** 10.7759/cureus.100132

**Published:** 2025-12-26

**Authors:** Jacob C Ball, Garrett J Rutt, Marcia Ballantyne

**Affiliations:** 1 Medicine, Lake Erie College of Osteopathic Medicine, Bradenton, USA; 2 Pathology, Lake Erie College of Osteopathic Medicine, Bradenton, USA

**Keywords:** agent orange exposure, hyponatremia, hyponatremia in icu, non small cell lung cancer, syndrome of inappropriate secretion of antidiuretic hormone (siadh)

## Abstract

We present the case of a 77-year-old male with a significant smoking history and prior Agent Orange exposure, who was admitted to the hospital multiple times with clinical and laboratory findings consistent with the syndrome of inappropriate anti-diuretic hormone (SIADH). The patient presented to the hospital on multiple occasions with symptoms and laboratory findings supportive of SIADH in the setting of malignancy. During his first visit, imaging studies demonstrated a right-sided pleural effusion, which was drained and sent for cytology and immunohistochemical staining studies. Following correction of his presenting hyponatremia, he was discharged with appropriate medications and follow-ups to discuss pleural fluid results. Three weeks later, he presented with similar symptoms, and the available pathology results of the pleural fluid specimen supported non-small cell lung cancer (NSCLC), likely adenocarcinoma.

This report highlights a rare occurrence of SIADH in the setting of NSCLC and emphasizes the importance of considering atypical paraneoplastic syndrome presentations in patients with multifactorial hyponatremia. It also engages in a discussion of the pathophysiology, clinical presentation, and diagnostic approach to SIADH in the context of NSCLC.

## Introduction

The syndrome of inappropriate antidiuretic hormone secretion (SIADH) is characterized by the excessive retention of free water due to unregulated release of antidiuretic hormone (ADH, or arginine vasopressin). Diagnosis is typically based on the presence of euvolemic hypotonic hyponatremia, decreased serum osmolality, elevated urine osmolality, and elevated urine sodium (>40 mEq/L), in the absence of clinical signs of hypovolemia or edema. SIADH has a broad range of etiologies, including central nervous system disturbances (e.g., stroke, hemorrhage, trauma), medications (cyclophosphamide, carbamazepine, and SSRIs), HIV, surgical stress, and malignancies. Among malignancies, ectopic production of ADH is most commonly associated with small cell lung cancer (SCLC). SIADH has also been reported in association with head and neck cancers, olfactory neuroblastoma, and extrapulmonary small cell carcinomas. However, SIADH is a rare finding in the context of non-small cell lung cancer (NSCLC) [1].

Historically, the management of SIADH focused primarily on identifying and addressing the underlying cause; however, at present, treatment regimens emphasize an individualized approach that considers the etiology, severity of hyponatremia, as well as the patient’s symptoms and volume status. For malignancy-associated SIADH, vasopressin receptor antagonists such as tolvaptan and conivaptan have shown favorable outcomes [2].

A comprehensive review of the literature using PubMed, Google Scholar, and UpToDate revealed only four published cases [3-5] of SIADH associated with NSCLC since 2004. Notably, no reports were found describing SIADH in patients with prior exposure to Agent Orange, a herbicidal chemical extensively used during the Vietnam War. Although Agent Orange exposure is a relevant element of this patient’s medical history, any potential association with SIADH remains speculative. The present case is therefore particularly uncommon, as it involves SIADH occurring in the context of NSCLC in a patient with documented Agent Orange exposure. Additionally, we review the diagnostic evaluation and management of severe hyponatremia (serum sodium <120 mmol/L), highlighting the need to recognize and evaluate atypical clinical presentations.

## Case presentation

**Figure 1:**
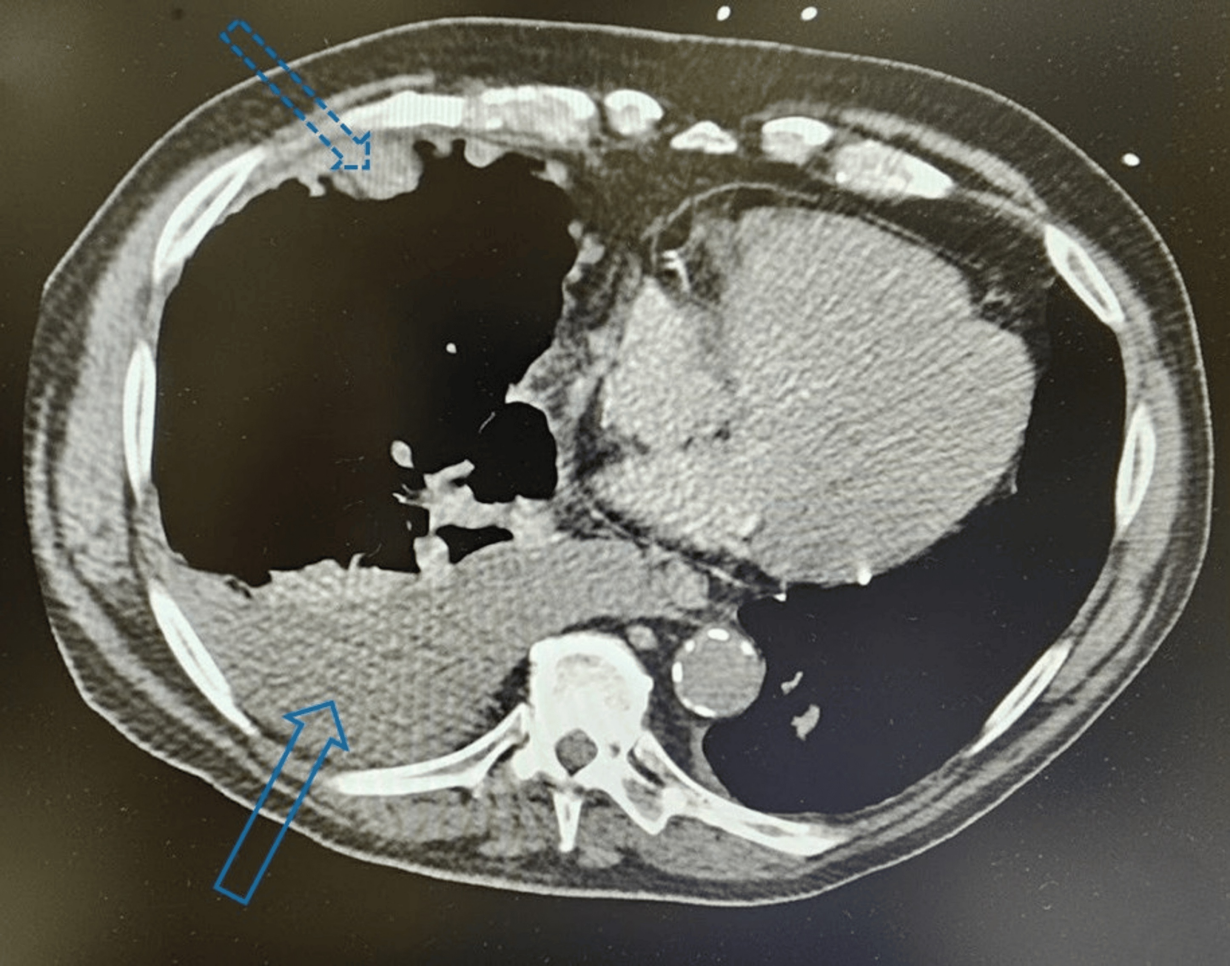
CT scan showing right pleural effusion The solid blue arrow at the bottom of the screen shows a significant pleural effusion in the right pleural space. The dotted blue arrow at the top of the screen displays peripheral nodularity CT: computed tomography

A 77-year-old male with a past medical history significant for coronary artery disease status post-coronary artery bypass graft x3, chronic obstructive pulmonary disease, gastroesophageal reflux disease, hyperlipidemia, hypertension, and history of pulmonary embolism due to hypercoagulable state presented to the emergency department for the first time with complaints of shortness of breath with nonproductive cough, blurred vision, fatigue, diarrhea, generalized weakness, and intermittent abdominal pain. Initial workup was remarkable for a serum sodium of 115 mmol/L, decreased serum osmolality of 213 mOsm/kg, elevated copeptin of 159.2 pmol/L, and decreased thyroid-stimulating hormone (TSH) of 0.373 mIU/L. Chest CT showed multiple lobulated polyploid pleural masses and a moderately sized right-sided pleural effusion, concerning for malignant effusion (Figure 1). After the patient’s hyponatremia was appropriately corrected with fluid restriction and urea sodium 15 g, he was discharged home and advised to follow up with nephrology for long-term management and oncology for biopsy of the lung nodule.

Three weeks later, the patient presented to his primary care physician complaining of increased weakness and fatigue. At this point, the patient was found to be hyponatremic, and due to his recent hospitalization, he was advised to return to the emergency department for further evaluation. Upon admission, labs were significant for a serum sodium of 110 mmol/L and serum osmolality of 236 mOsm/kg. 

Due to the severity of his hyponatremia, the patient was transferred to the ICU for closer monitoring and management. He was started on a continuous infusion of 3% hypertonic saline at 20 mL/hour. Oral urea-sodium was later supplemented to address refractory hyponatremia at a goal correction rate of 6-8 mEq/L over 24 hours. During this second hospitalization, the patient’s serum sodium fluctuated significantly. It initially improved from a low of 110 mmol/L to 123 mmol/L, accompanied by a low 24-hour urine output of approximately 200 mL. These findings of persistent hyponatremia with variable urine output suggested a mixed etiology, possibly involving underlying volume depletion in addition to SIADH. Given the inadequate response to fluid restriction, hypertonic saline, and urea-sodium, further pharmacologic intervention was deemed necessary. Tolvaptan, a vasopressin receptor antagonist, was initiated to promote diuresis and correct the resistant hyponatremia. This choice was preferred over desmopressin due to the running diagnosis of SIADH secondary to malignancy. 

Over the following days, the patient was closely monitored, and adjustments were made to the administration of hypertonic saline and tolvaptan to maintain appropriate serum sodium levels and to avoid hyponatremia. By hospital day three, his serum sodium had increased to 128 mmol/L, and the patient was no longer symptomatic. Additionally, the patient’s repeated lab workup revealed a rising N-terminal pro-brain natriuretic peptide (proBNP), reaching 1600 pg/mL. Due to this finding, empagliflozin, an SGLT2 inhibitor, was added to the treatment regimen to assist with volume management secondary to potential underlying heart failure.

**Figure 2:**
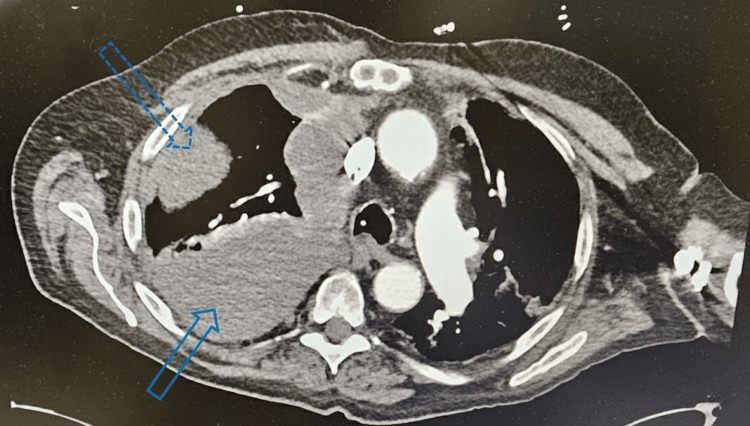
CT scan showing increased right pleural effusion with peripheral pleural changes The solid blue arrow at the bottom of the screen points towards increased right pleural effusion posteriorly versus previous CT scans. The dotted blue arrow at the top of the screen shows an increasingly significant peripheral change versus previous CT scans CT: computed tomography

By day four, the patient’s serum sodium had reached 131 mmol/L. He was transferred to the intensive care step-down unit in stable condition with instructions to continue empagliflozin, urea-sodium 15 g daily, and follow up with interventional radiology for further evaluation. A CT scan showed increased right pleural effusion with peripheral nodular changes (Figure 2). A right-sided thoracentesis was performed, which demonstrated malignant cells in clusters consistent with NSCLC with a strong likelihood of adenocarcinoma. The malignant cells tested positive for CK7, CEA, and TTF1, but negative for CK20, CDX2, and INSM1 on immunohistochemical staining. The patient was promptly informed of his stage IV lung cancer diagnosis based on his clinical findings, CT results, and cytological and immunohistochemical markers.

The patient was strongly advised to follow up with his primary care physician and oncology team for further management and treatment planning. His fluids were continually managed in the step-down unit as his serum sodium stabilized around 135 mmol/L and serum osmolality of 260 mOsm/kg. A CT-guided biopsy and plans for chemotherapy were discussed, but were unable to be completed due to the patient’s rapid clinical deterioration in conjunction with recurrent pleural effusions. Unfortunately, the patient passed away a few weeks later before obtaining a solid tissue biopsy to confirm the diagnosis of adenocarcinoma. A summary of the patient's pertinent labs in the context of Schwartz and Bartter's SIADH diagnostic criteria during his hospital course is presented in Table 1 [6].

**Table 1:** Electrolyte and marker trends *Copeptin is a marker related to ADH production; "-": not measured N-terminal proBNP: N-terminal fragment of pro-brain natriuretic peptide; TSH: thyroid-stimulating hormone

Parameters	Reference range	SIADH diagnostic criteria	Initial hospitalization	Second hospitalization day 1	Second hospitalization day 2	Second hospitalization day 3	Second hospitalization day 4
Serum sodium (mmol/L)	135-145	<135	115	110	123	128	131
Serum osmolality (mOsm/kg)	275-295	<275	213	236	245	248	258
Urine sodium (mmol/L)	20-50	>40	<30.0	-	-	-	-
Urine osmolality (mOsm/kg)	50-1200	>100	559	-	-	-	-
N-terminal proBNP (pg/mL)	0-300	-	736	-	-	1488	1600
Copeptin^*^ (pmol/L)	1.70-11.25	-	159.2	-	-	-	-
TSH (mIU/L)	0.340-5.60	-	0.373	-	-	-	-

## Discussion

According to the National Cancer Institute, the estimated incidence of lung cancer in 2022 was 51 per 100,000 among men and 43.7 per 100,000 among women in the United States [7]. Multiple risk factors contribute to lung cancer development, including radon, asbestos, silica, radiation exposure, and most notably, cigarette smoking. Cigarette smoke is implicated in 80-90% of lung cancer-related deaths [8]. Paraneoplastic syndromes occur in approximately 1-7.4% of all cancer patients and are defined as tumor secretion of hormones, peptides, or immune-mediated responses, rather than from direct tumor invasion or metastasis [9]. Lung cancers, especially small-cell lung cancer (SCLC), are the most commonly associated malignancies. SCLC accounts for about 15% of all lung cancer cases and is characterized by its aggressive clinical course and high mitotic activity [10].

SCLC is associated with both hormone-mediated and immune-mediated paraneoplastic syndromes. Common endocrine syndromes include ectopic Cushing’s syndrome (5% incidence) and SIADH-related hyponatremia (15% incidence). Less frequently, patients may develop Lambert-Eaton myasthenic syndrome, hypertension, or amenorrhea [10]. In contrast, the most frequently observed paraneoplastic syndrome in NSCLC is hypercalcemia due to parathyroid hormone-related peptide (PTHrP) secretion in squamous cell carcinoma [11]. SIADH remains a rare finding in NSCLC, with only four documented cases [3-5] reported in the literature since 2004.

Hyponatremia is a common clinical challenge that is often multifactorial in origin. In the context of malignancy, a diagnosis of SIADH must be made after carefully excluding other potential causes. Symptoms of hyponatremia can range from mild (nausea, headache, fatigue) to severe (confusion, seizures, coma). Treatment of SIADH aims primarily at addressing the underlying cause. In cases of malignancy, tumor resection or chemotherapy may be curative; however, management of the patient’s symptoms should also occur. In symptomatic cases, hypertonic saline is recommended for prompt correction. For asymptomatic patients, fluid restriction (initially 500 mL/day) and increased solute intake are preferred initial strategies [12].

In cases where first-line therapy fails, as in our patient, second-line treatments such as oral urea-sodium and vasopressin receptor antagonists (e.g., tolvaptan, conivaptan) may be necessary. It is important to ensure that the correction of hyponatremia does not occur too rapidly, particularly in chronic cases (>48 hours duration or progression <0.5 mmol/hr), as rapid increases in serum sodium increase the patient’s risk of osmotic demyelination syndrome (ODS), formerly known as central pontine myelinolysis. This life-threatening condition results from a rapid osmotic shift leading to cellular dehydration and demyelination within the central nervous system [13]. To mitigate this risk, current guidelines recommend limiting correction to no more than 6-8 mmol/L within 24 hours.

As outlined in the case description, the patient was prescribed empagliflozin in response to rising proBNP levels, a marker commonly elevated in heart failure. Empagliflozin was given to increase fluid offloading in the setting of impending heart failure. In cases of SIADH, the body initiates a physiological response known as vasopressin escape, an adaptive mechanism, supported in part by aldosterone, that prevents ongoing serum sodium dilution despite persistently elevated plasma vasopressin [14]. This process typically results in a urine sodium concentration exceeding 40 mmol/L. However, in hypervolemic states such as heart failure, nephrotic syndrome, or cirrhosis, urinary sodium excretion is often below 20 mmol/L. Given the patient’s cardiac history, this blunted sodium excretion is consistent with volume overload secondary to heart failure.

The patient discussed in this case had experienced significant Agent Orange exposure during previous military service. Agent Orange was an herbicide used by the United States military to destroy vegetation for tactical advantages against the Vietnamese. Agent orange is a known toxin and has been associated with an increased risk of various medical conditions, including but not limited to idiopathic pulmonary fibrosis, bladder cancer, and dementia [15-17].

As illustrated in this report, SIADH can occur outside of its typical association with SCLC. This report contributes to the existing literature that demonstrates atypical presentations of paraneoplastic syndromes in the setting of NSCLC, reinforcing the need for clinicians to maintain a high index of suspicion when evaluating unexplained hyponatremia [3-5]. The patient’s history of Agent Orange exposure further adds to the clinical complexity, although a direct link between this exposure and the development of SIADH remains speculative.

## Conclusions

To the best of our knowledge, this is the first documented case of SIADH associated with NSCLC, likely adenocarcinoma, in a patient with a history of Agent Orange exposure. While a direct causal link between Agent Orange and the development of NSCLC cannot be definitively established, the patient's unique exposure history adds an important dimension to the case and warrants further investigation. The patient was admitted twice with consistent symptoms, laboratory findings, imaging, cytology, and immunohistochemical staining profiles, and treatment responses that strongly supported the diagnosis of SIADH in the context of NSCLC. Although SIADH is a well-recognized paraneoplastic syndrome, its occurrence in NSCLC remains exceedingly rare, with very few cases reported in the literature. This report highlights the importance of maintaining a broad differential and recognizing that paraneoplastic syndromes may not follow a typical presentation. It also emphasizes the value of a thorough diagnostic workup and patient history, even when initial findings deviate from textbook teachings.
